# Effects of Moderate–Intensity Physical Training on Skeletal Muscle Substrate Transporters and Metabolic Parameters of Ovariectomized Rats

**DOI:** 10.3390/metabo12050402

**Published:** 2022-04-29

**Authors:** Taciane Maria Melges Pejon, Pedro Paulo Menezes Scariot, Heloísa Sobreiro Selistre-de-Araujo, Claudio Alexandre Gobatto, Anabelle Silva Cornachione, Wladimir Rafael Beck

**Affiliations:** 1 Laboratory of Endocrine Physiology and Physical Exercise, Department of Physiological Sciences, Federal University of São Carlos, São Carlos 13565-905, São Paulo, Brazil; tacianepejon92@gmail.com; 2 Laboratory of Applied Sport Physiology, Department of Sport Sciences, School of Applied Sciences, State University of Campinas, Limeira 13484-350, São Paulo, Brazil; pedroppms@yahoo.com.br (P.P.M.S.); claudio.gobatto@fca.unicamp.br (C.A.G.); 3 Laboratory of Biochemistry and Molecular Biology, Department of Physiological Sciences, Federal University of São Carlos, São Carlos 13565-905, São Paulo, Brazil; hsaraujo@ufscar.br; 4 Muscle Physiology and Biophysics Laboratory, Department of Physiological Sciences, Federal University of São Carlos, São Carlos 13565-905, São Paulo, Brazil; cornachione@ufscar.br

**Keywords:** estrogen, GLUT4, FAT CD36, energetic balance, intermediary metabolism, physical exercise, hypoestrogenism, lipid metabolism, carbohydrate metabolism

## Abstract

A deficit of estrogen is associated with energy substrate imbalance, raising the risk of metabolic diseases. Physical training (PT) is a potent metabolic regulator through oxidation and storage of substrates transported by GLUT4 and FAT CD36 in skeletal muscle. However, little is known about the effects of PT on these carriers in an estrogen-deficit scenario. Thus, the aim of this study was to determine the influence of 12 weeks of PT on metabolic variables and GLUT4 and FAT CD36 expression in the skeletal muscle of animals energetically impaired by ovariectomy (OVX). The trained animals swam 30 min/day, 5 days/week, at 80% of the critical load intensity. Spontaneous physical activity was measured biweekly. After training, FAT CD36 and GLUT4 expressions were quantified by immunofluorescence in the soleus, as well as muscular glycogen and triglyceride of the soleus, gluteus maximus and gastrocnemius. OVX significantly reduced FAT CD36, GLUT4 and spontaneous physical activity (*p* < 0.01), while PT significantly increased FAT CD36, GLUT4 and spontaneous physical activity (*p* < 0.01). PT increased soleus glycogen, and OVX decreased muscular triglyceride of gluteus maximus. Therefore, OVX can cause energy disarray through reduction in GLUT4 and FAT CD36 and their muscle substrates and PT prevented these metabolic consequences, masking ovarian estrogen’s absence.

## 1. Introduction

Estrogens are a group of feminizing steroid hormones secreted in significant amounts by the ovaries, estradiol being the most abundant. Although classically recognized by reproductive hormonal action [1,2], estrogens are involved in a broad metabolic capacity such as in energy balance modulation, thermogenesis stimulation [3], and glycemic [4] and lipidic regulation [5,6]. The abrupt decline of estrogens is commonly associated with metabolic disorder, triggered by an imbalance of energy usage and consumption [7,8]. This explains why menopause or bilateral surgical removal of the ovaries are contributing factors for the development of obesity [9], diabetes mellitus [4,7], dyslipidemia [5,9] and cardiovascular disease [10,11].

Directing the theme of energy regulation to a tissue that plays a crucial role in such scenario, it is noteworthy that skeletal muscle, corresponding to 40% of body mass, is one of the major elements involved in energy homeostasis mainly by the uptake of fatty acids [12] and glucose [13], consumed to generate energy or stored as triacylglycerol [5] and glycogen [14]. In skeletal muscle, glucose and fatty acid uptake are dependent mainly on glucose transporter 4 (GLUT4) [13,15] and fatty acid translocase CD36 (FAT CD36) [5,16], respectively. Both transporters are translocated to sarcolemma via insulin-dependent signaling [4,16], a pathway also stimulated by estrogens [13,17]. Skeletal muscle contains many alpha (ERα) and beta estrogen receptors (ERβ); however, the literature presents contradictory information regarding the influence of estrogen deficit on GLUT4 levels [18,19,20], while few studies address FAT CD36. ERα knockout results in a reduction in lipid oxidation in skeletal muscle [21], though without ascertaining what happens to FAT CD36. Thus, molecular knowledge is crucial to understanding tissue functioning and subsequent systemic metabolic imbalance in this context. Despite these existing gaps, the numerous disadvantages that hypoestrogenism causes on energy metabolism are well established [4,5,7,9,11,22], requiring methods that act as prevention of it without causing undesirable side effects.

Physical exercise is one of those applicants, modulating skeletal muscle energy intake in response to both muscle contraction and activation of the insulin pathway [15,23]. Both processes result in translocation of GLUT4 [15] and FAT CD36 [5] with consequent glucose and fatty acid uptake, respectively. Physical training (PT) induces the synthesis of GLUT4 [15,23], favoring glucose uptake and glycogen replacement [13,23]. Regarding FAT CD36, aerobic physical training is known to increase fatty acid consumption [23]; thus, the activation of FAT CD36 in the supply of fatty acids for the oxidation process is essential [5]. However, despite the positive role of physical training concerning glycolytic [15] and plasma lipid regulation [24], the literature does not provide consolidated answers about its effect on skeletal muscle GLUT4 and FAT CD36 in the absence of ovarian estrogens. Thus, the aim of the present study is to determine the impact of 12 weeks of moderate-intensity PT on energy parameters and GLUT4 and FAT CD36 expressions through immunofluorescence in the skeletal muscle of animals energetically impaired by ovarian estrogen deficit. We hypothesize that the proposed swimming PT will prevent the undesirable metabolic effects caused by ovariectomy, preventing a reduction in GLUT4 and FAT CD36.

## 2. Results

### 2.1. FAT CD36 Quantification by Immunofluorescence 

**Figure 1:**
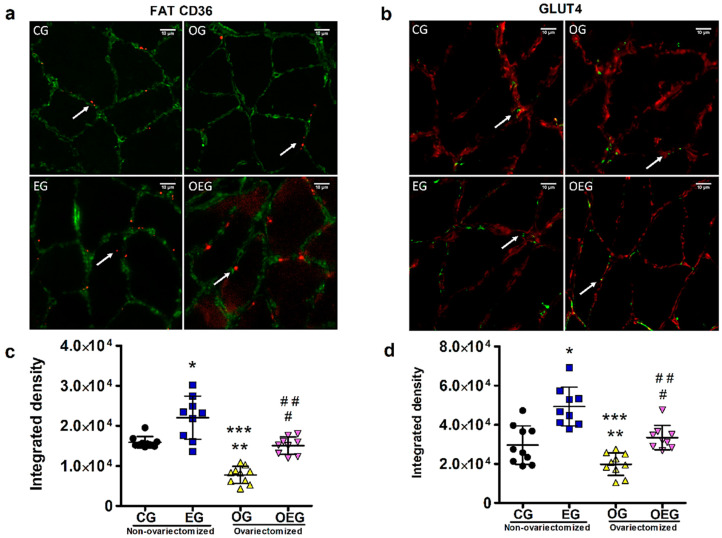
Representative immunofluorescence images from tissue sections: (**a**) laminin (green) and FAT CD36 (red) in soleus skeletal muscle and (**b**) laminin (red) and GLUT4 (green) in soleus skeletal muscle in the control group (CG; *n* = 10), ovariectomized group (OG; *n* = 10), exercised group (EG; *n* = 9), and ovariectomized and exercised group (OEG; *n* = 9). The white arrows indicate the proteins, FAT CD36 in the panel (**a**) and GLUT4 in the panel (**b**). Graphs represent the average from integrated intensity values of FAT CD36 (**c**) and GLUT4 (**d**) expressions obtained from each animal. Data are represented as mean and standard deviation. The statistics provided in the graphs are the results of the Newman–Keuls post hoc test: * *p* < 0.05 EG in relation to CG; ** *p* < 0.05 OG in relation to CG; *** *p* < 0.05 OG in relation to EG; # *p* < 0.05 OEG in relation to EG; ## *p* < 0.05 OEG in relation to OG. For illustrative images objective lens = 20x was used; scale bar = 10 µm.

OVX decreased FAT CD36 (F = 56.01; *p* < 0.01) and PT induced an increase (F = 43.98; *p* < 0.01), but there was no interaction (F = 0.35; *p* = 0.55). There was no difference between OEG and CG (*p* = 0.55), as seen in Figure 1a,c.

### 2.2. GLUT4 Quantification by Immunofluorescence

OVX promoted a decrease in GLUT4 (F = 23.43; *p* < 0.01), while PT promoted an increase (F = 39.28; *p* < 0.01), but without interaction (F = 1.32; *p* = 0.25). There was no difference for OEG in relation to CG (*p* = 0.32), as seen in Figure 1b,d.

### 2.3. Muscular Glycogen, Muscular Triglyceride and Blood Glucose

**Figure 2:**
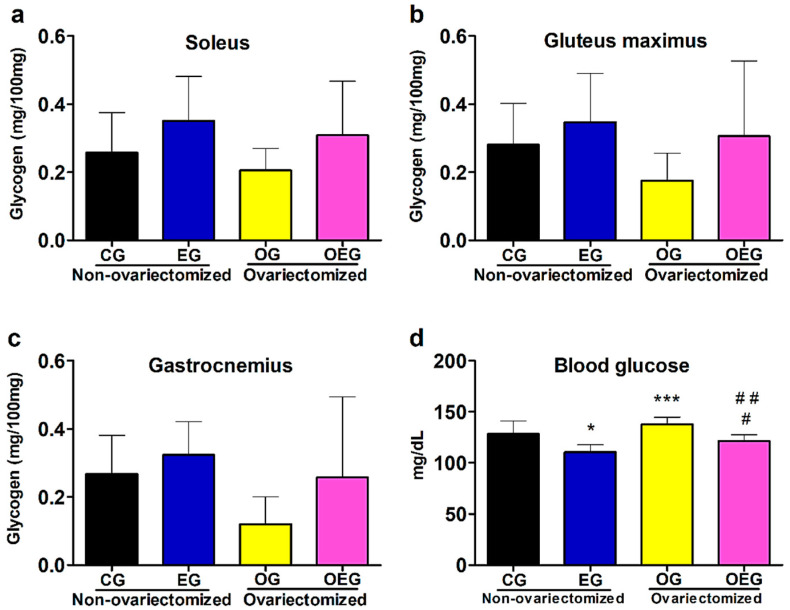
Data from muscular glycogen of the soleus (**a**), gluteus maximus (**b**) and gastrocnemius (**c**), as well as blood glucose (**d**) in the control group (CG; *n* = 10), ovariectomized group (OG; *n* = 10), exercised group (EG; *n* = 9) and ovariectomized and exercised group (OEG; *n* = 9). Values expressed as mean and standard deviation. The statistics provided in the graphs are the results of the Newman–Keuls post hoc test: * *p* < 0.05 EG in relation to CG; *** *p* < 0.05 OG in relation to EG; # *p* < 0.05 OEG in relation to EG; ## *p* < 0.05 OEG in relation to OG. mg: milligrams; dL: deciliters.

As for muscular glycogen, PT promoted a significant increase in the soleus (F = 5.66; *p* < 0.05), but OVX did not promote a significant difference (F = 1.31; *p* = 0.26) and there was no verified interaction (F = 0.01; *p* = 0.90). PT and OVX did not promote a difference in the gluteus maximus (F = 3.04, *p* = 0.09 and F = 1.67, *p* = 0.20, respectively) and there was no interaction (F = 0.32; *p* = 0.57). For the gastrocnemius, PT and OVX also had no significant effect (F=3.18, *p* = 0.08 and F = 3.91, *p* = 0.06, respectively) and no interaction (F = 0.56; *p* = 0.45) (Figure 2a–c).

PT responses showed a significant decrease in blood glucose (F = 27.66; *p* < 0.01), while OVX significantly increased it (F = 9.57; *p* < 0.01), but there was no significant interaction of the effects (F = 0.06; *p* = 0.80) (Figure 2d).

**Figure 3:**
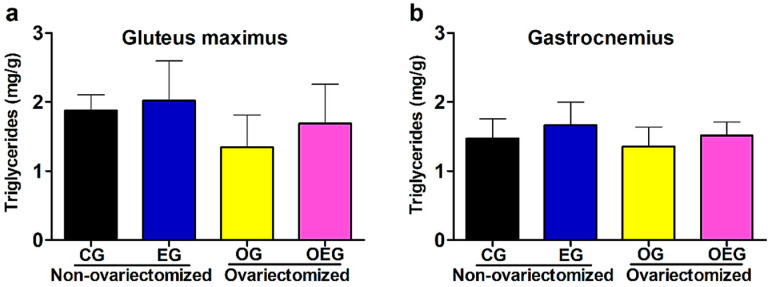
Data from muscular triglyceride of the gluteus maximus (**a**) and gastrocnemius (**b**) in the control group (CG; *n* = 10), ovariectomized group (OG; *n* = 10), exercised group (EG; *n* = 9) and ovariectomized and exercised group (OEG; *n* = 9). Values expressed as mean and standard deviation. mg: milligrams; g: grams.

In relation to muscular triglyceride, PT did not promote a significant difference in the gluteus maximus (F = 1.71; *p* = 0.20) and gastrocnemius (F = 2.87; *p* = 0.10). However, OVX promoted a significant reduction in the gluteus maximus (F = 5.27; *p* < 0.05) but did not promote significant alteration in the gastrocnemius (F = 1.70; *p* = 0.20). For both muscle tissues, interaction was not verified (F = 0.28, *p* = 0.59 for gluteus maximus and F = 0.02, *p* = 0.88 for gastrocnemius) (Figure 3a,b).

### 2.4. Body Mass and Spontaneous Physical Activity

**Figure 4:**
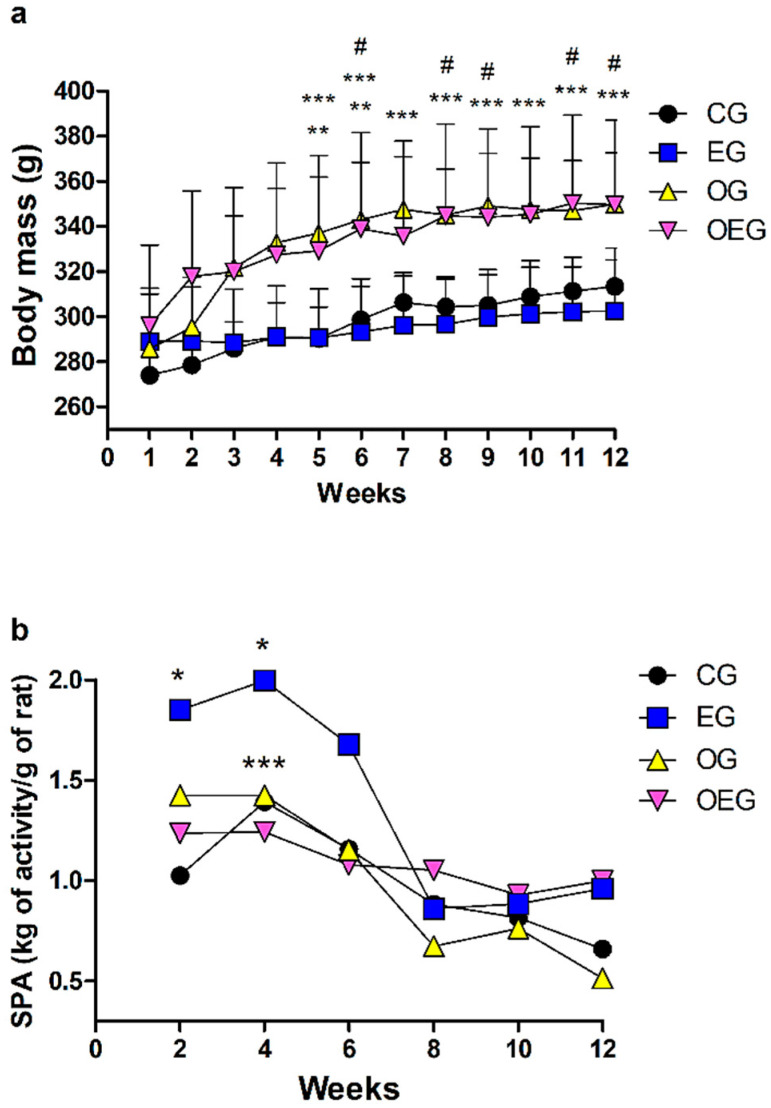
Data from body mass (**a**) and spontaneous physical activity (**b**) throughout 12 weeks of experiment in the control group (CG; *n* = 10), ovariectomized group (OG; *n* = 10), exercised group (EG; *n* = 9) and ovariectomized and exercised group (OEG; *n* = 9). Values expressed as mean and standard deviation for body mass and as sum of the day for spontaneous physical activity. The statistics provided in the graphs are the results of the Newman–Keuls post hoc test: * *p* < 0.05 EG in relation to CG within same week; ** *p* < 0.05 OG in relation to CG within same week; *** *p* < 0.05 OG in relation to EG within same week; # *p* < 0.05 OEG in relation to EG within same week. SPA: spontaneous physical activity; kg: kilograms; g: grams.

The initial body mass (g) mean and standard deviation of animals not submitted to ovariectomy was 281 ± 20, while animals submitted to PT was 290 ± 30 (*p* > 0.05). At the end of the 12-week intervention, the non-ovariectomized animals presented a mean and standard deviation of 308 ± 21, while those submitted to ovariectomy ended with a mean and standard deviation of 349 ± 30 (increase of 13%; *p* < 0.01) (Figure 4a). Over the 12 weeks, OVX and time resulted in a significant increase in body mass (F = 216.2; *p* < 0.01 and F = 10.1; *p* < 0.01, respectively). PT did not promote a significant difference in the body mass (F = 0.40; *p* = 0.52).

PT promoted an increase in spontaneous physical activity (F = 19.75; *p* < 0.01) over the 12-week period of the experiment. OVX reduced spontaneous physical activity (F = 6.67; *p* < 0.05). The time (main effect) reduced spontaneous physical activity (F = 22.7; *p* < 0.01) and a significant interaction was verified (F = 4.89; *p* < 0.01). In the second and fourth weeks, there were a significant increase in spontaneous physical activity of the EG compared to the CG (*p* < 0.05). From the eighth week, all groups saw a decline in spontaneous physical activity, with no significance among them (Figure 4b).

## 3. Discussion

According to the findings of this study, an estrogen deficit metabolically impairs skeletal muscle, tissue with a considerable number of estrogenic receptors and which is very important in energy homeostasis. However, 12 weeks of moderate-intensity swimming training avoided harmful changes in metabolic parameters of skeletal muscle, such as GLUT4 and FAT CD36 expressions.

Regarding carbohydrate metabolism, OVX promoted a reduction in GLUT4 in the soleus skeletal muscle (F = 23.43; *p* < 0.01), possibly influencing the increase in the blood glucose by OVX (F = 9.57; *p* < 0.01) due to GLUT4′s lower responsiveness to uptake circulating glucose. To try to understand our result, a study using an ERα agonist resulted in glucose uptake by stimulating the Akt (Akt) and Akt substrate (AS160), leading to the phosphorylation of AMP-activated protein kinase (AMPK) [25]. The activation of Akt and AMPK conditions the phosphorylation of the AS160, initiating the translocation of GLUT4 to the cell membrane in order to capture glucose [26], demonstrating the role of estrogens in glycolytic homeostasis [4,13,25,26,27]. Instead, a study using an estrogen antagonist found a reduction in AMPK activation [17]. However, the literature presents inconclusive results, possibly associated with the period of absence of ovarian estrogens. Saengsirisuwan et al. [20] found a reduction in GLUT4 after 12 weeks of OVX, while MacDonald et al. [19], Hansen et al. [28] and Chen et al. [18] did not find a significant reduction in GLUT4 at 4, 6 and 8 weeks after OVX, respectively. In summary, changes in GLUT4 expression can induce insulin resistance [29]. Lower GLUT4 expression reduces glucose uptake by skeletal muscle, leading to lower glycogen storage [23] and reflecting an increase in blood glucose, which may result in an increased risk of severe comorbidities.

As a preventive method on the molecular impairment found, PT can be an exceptional tool due to the metabolic adaptations generated. This study opted for swimming, considering that rats have the natural ability to swim [30], besides being a less physically traumatic exercise and with a greater possibility of verifying a pattern of activity among animals [31]. Another important factor is due to the high muscle recruitment of the soleus, gluteus maximus and gastrocnemius during swimming exercise [32]. When considering the metabolic point of view of physical training applied as being predominantly aerobic due to moderate intensity, it was decided to choose the soleus muscle for the evaluation of the main responses generated due to its composition of 87% oxidative fibers [33].

When inserting PT, the training duration in weeks has also been shown to be an important factor to promote positive changes in GLUT4 expression after OVX. Saengsirisuwan et al. [20] and Hansen et al. [28] found an increase in GLUT4 after running and swimming, respectively, while MacDonald et al. [19] were unsuccessful after 4 weeks. These comparisons justify our 12-week PT choice, in which we verified an increase in GLUT4 expression employing swimming (F = 39.28; *p* < 0.01), increase in glycogen in the soleus muscle (F = 5.66; *p* < 0.05) and a reduction in the blood glucose (F = 27.66; *p* < 0.01). This increase in GLUT4 is responsible for the proportional entry of glucose, being fundamental for the process of glycogen overcompensation after the exercise [13,23], and blood glucose homeostasis. It is known that muscle contraction increases the cytosolic concentration of Ca^2^^+^ calmodulin/CaMK [34,35], activating important regulators such as AMPK [34], peroxisome proliferator-activated receptor beta (PPARβ) and peroxisome proliferator-activated receptor gamma coactivator 1 alpha (PGC-1α), resulting in increased glucose uptake by stimulating the synthesis and activity of GLUT4 [15]. The literature also systematically shows that the sensitivity of insulin action is also improved by physical training, influencing GLUT4 activity [13]. Therefore, considering the results obtained in this study, PT promoted increased GLUT4 and muscle glycogen while reducing blood glucose despite the energetic adversity of hypoestrogenism, contributing positively to the prevention of diseases associated with the imbalance of carbohydrate metabolism.

Regarding lipid metabolism, OVX promoted a reduction in the FAT CD36 expression (F = 56.01; *p* < 0.01), which was in line with the reduced muscular triglyceride in the gluteus maximus for ovariectomized groups (F = 5.27; *p* < 0.05). The reduction of fatty acid uptake due to changes in FAT CD36 expression negatively affects the transport and oxidation of these substrates in muscle tissue, helping in parts in understanding the relationship of lipid imbalance and hypoestrogenism. In such scenario, the muscular triglyceride reduction could impair the skeletal muscle intracellular energy production [36] due to the predominant use of lipids at rest and low to moderate physical training intensity [37]. Although the present study did not evaluate it, peroxisome proliferator-activated receptor alpha (PPARα) is reduced in ovariectomized animals [38], as well as PGC-1α [39], being established that PPARα [38] and PGC-1α [40] promote upregulating FAT CD36 expression.

When ovariectomized animals were submitted to 8 weeks of running PT, Kim et al. [5] reported a subtle but not significant increase in FAT CD36. We found an increase in FAT CD36 (F = 43.98; *p* < 0.01). Seeking to understand this result, through muscle contraction, the translocation of FAT CD36 can occur via AMPK [16,41] and via CaMK responsible for regulating PGC-1α [42], a factor known for promoting FAT CD36 upregulation [43]. Furthermore, PGC-1α interacts with PPARs [12], being the beta isoform (PPARβ) highly expressed protein in aerobic training and responsible for increased FAT CD36 synthesis [16], which may increase the ability to oxidize fatty acids [12].

We considered some principles of the Randle cycle regarding the results of our study. Although we applied aerobic training with high demand on the oxidative pathway from lipids during exercise, the use of glycogen was fundamental for maintaining the Krebs cycle [44], justifying the importance of raising the levels of this substrate in muscle tissue, as well as increased GLUT4, as observed in this study. These theoretical approaches can contribute to the understanding of the increase in FAT CD36 and GLUT4 in view of the energy demand imposed in response to physical training, including being relevant for the muscle recovery period. In this sense, although proteins were evaluated in a highly oxidative muscle such as soleus, this result allows to subtly deduce that other muscles similarly recruited during this physical training protocol would present equivalent responses in relation to FAT CD36 and GLUT4 expression. Interestingly, PT also exerted a positive influence on the energetic context of OEG animals, which are metabolically impaired by estrogen deficit, allowing similarity in the skeletal muscle parameters when compared to CG animals.

The energy balance is another important parameter commonly influenced by ovariectomy [3]. We found a significant spontaneous physical activity reduction by OVX (F = 6.67; *p* < 0.05), corroborating with Sherk et al. [45], after 11 weeks. Although not elucidated knowledge, it is possible that estrogens can interfere with the total volume of daily spontaneous physical activity by compromising the action of orexin, a neuropeptide synthesized in the perifornical region, which also expresses estrogen receptors [46]. Such reduction in the daily spontaneous physical activity is reflected in the increased body mass [47], favoring obesity [48] and other metabolic diseases due to lower energy expenditure and lower metabolic rate [7]. On the other hand, considering the role of exercise in stimulating energy expenditure [49], we verified that PT was efficient in raising spontaneous physical activity (F = 19.75; *p* < 0.01). As physical activity is characterized by any movement that leaves the resting condition, this action favors energy expenditure primarily by causing the stimulation of skeletal muscle with consequent energy consumption [50].

**Figure 5:**
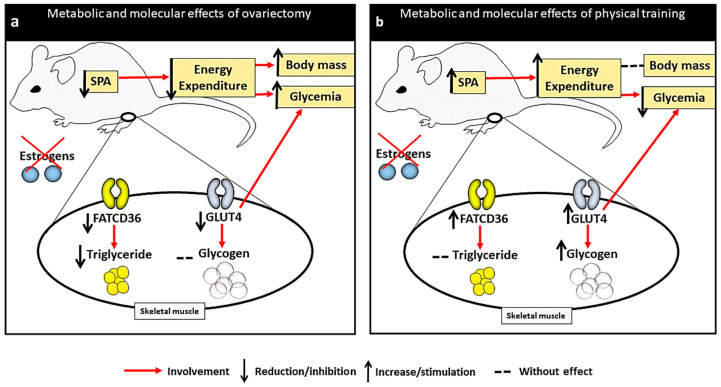
Illustration of the statistical effects (factorial ANOVA) of ovariectomy and physical training on metabolic and molecular parameters investigated in this study. (**a**) Representation of the ovariectomy effect causing reduction in the variables spontaneous physical activity, GLUT4 (soleus), FAT CD36 (soleus) and muscular triglyceride (gluteus maximus) with consequent increase in blood glucose and body mass. (**b**) Representation of the effect of physical training in ovariectomized rats preventing most of these metabolic impairments by increasing of the spontaneous physical activity, GLUT4 (soleus), FAT CD36 (soleus) and glycogen (soleus) with consequent reduction in blood glucose. SPA: spontaneous physical activity.

Our data set demonstrated the effectiveness of PT in preserving metabolic changes that may be harmful, despite the lack of ovarian estrogens (Figure 5). These findings show that a PT considered to be of moderate domain [51], such as that which we applied, was an excellent preventive method in this context, allowing animals with low estrogen concentration to present similar metabolic responses to animals with physiological levels of estrogens, in order to avoid metabolic disorders.

In summary, a lack of ovarian estrogen can affect energy balance by a network of factors that relate to tissue and serum parameters and spontaneous physical activity evaluated by this study. We found a reduction in daily physical activity, GLUT4 and FAT CD36 expression and muscle energy levels, as well as increased blood glucose. However, our 30 min daily protocol of swimming for 12 weeks at moderate intensity was efficient in nullifying some negative metabolic consequences of ovariectomy, especially in relation to the expression of GLUT4 and FAT CD36 and daily physical activity, acting as a compensator of the estrogen’s deficit in this context.

## 4. Material and Methods

### 4.1. Animals

We evaluated 38 female Wistar rats obtained from the central Bioterium of the Federal University of São Carlos and subsequently housed in the Bioterium of the Laboratory of Endocrine Physiology and Physical Exercise. The bioterium follows a light/dark cycle of 10/14 h, relative humidity between 45 and 55% and controlled temperature of 22 ± 2 °C, and the animals received specific feed for rodents (Presence^®^, Brazil) and water ad libitum throughout the experiment. The rats were collectively housed at the density of five animals per cage. The experimental procedure was conducted in accordance with the Guide for the Care and Use of Laboratory Animals, and maximum care was ensured to minimize the number of animals used and their suffering in this study. The experiment was approved by the Ethics Committee on Animal Use (CEUA) of the Federal University of São Carlos under protocol no. 1556060417.

### 4.2. Experimental Design

At 45 days old, the animals arrived at the bioterium for familiarization to the environment and were randomly distributed into four groups: control (CG: *n* = 10), ovariectomized (OG: *n* = 10), exercised (EG: *n* = 9) and ovariectomized and exercised (OEG: *n* = 9). At 90 days old, the OG and OEG animals were subjected to the surgical technique of bilateral ovariectomy (OVX). At 95 days old, EG and OEG initiated the aquatic environment adaptation, and at 102 days old, such groups were subjected to the first critical load test to determine the exercise intensity for physical training (PT), conducted over a 12-week period.

### 4.3. Surgical Procedure of Bilateral Ovariectomy

The technique was preceded with intraperitoneal anesthesia: ketamine (10 mg.kg^−1^) and xylazine (0.1 mg.kg^−1^) for the ovariectomized groups (OG and OEG). According to Zarrow et al. [52], a 1.5 cm incision was made on both sides in the skin, lateral to the spine, between the last rib and the knee, allowing the ovaries to be exposed, tied and removed from the pelvic cavity. The remainder of the tissue returned to the peritoneal cavity and all the layers were sutured with cotton thread (line 10). The animals of the OG and OEG groups were inspected daily in the post-surgical period and rested for 5 days. Since complications were not observed, all animals were able to proceed with aquatic adaptation.

### 4.4. Aquatic Environment Adaptation

The protocol of familiarization to the aquatic environment was adapted from the models proposed by Gobatto et al. [53] and De Lima et al. [54]. At 95 days old, the animals (EG and OEG) were subjected to 6 days of progressive adaptation, observing parameters such as exposure time in water of 5–20 min, a depth of 10–80 cm and load weight varying from 0 to 3% of body mass. The adaptation and physical training were performed in individual cylindrical tanks with an 80 cm column of water, 30 cm diameter, and temperature maintained at 31 ± 1 °C, following the guidelines of the American Physiological Society [31].

### 4.5. Critical Load Intensity Determination and Swimming Training Protocol

At 102 days old, the animals (EG and OEG) were subjected to the critical load test, which is based on mathematical analysis of the relationship between exercise intensity and time to achieve exhaustion. The critical load test involved performing 4 maximum-intensity tests with different loads to exhaustion times between 2 and 10 min. The time to exhaustion recorded in seconds was determined when the rats were unable to swim properly following criteria established by Beck and Gobatto [55]. The time and load data were subjected to analysis by linear regression based on the third model proposed by Gobatto et al. [53], and it was decided to accept a coefficient of determination (R^2^) higher than 0.95. The critical load (% body mass) was assumed as the angular coefficient from the linear fit. Regarding swimming training protocol, the animals swam with 80% of the individual critical load, with weekly weighing for load readjustment. The physical training was performed 30 min daily, 5 days a week over 12 weeks. The critical load test was reapplied in the fifth and ninth weeks for critical load intensity fine adjustment. The mean involving the 3 critical load intensities was 5.9% of body mass for the EG and 6.1% of body mass for the OEG.

### 4.6. Spontaneous Physical Activity

Spontaneous physical activity was measured biweekly using the gravimetric principle which has been employed by many investigators [56,57,58,59,60,61]. The principle of gravimetric apparatus is that movements generated by rats induce a force on the platform that can be quantified by load cells (MKPW^®^, MK Control and Instrumentation™, São Paulo, Brazil) as a change in weight. This system is imperceptible to the animal and consists of capturing the signals generated by a strain gauge in millivolts (mV). This signal passed through an amplifier (MKTC5-10^®^, MK Control and Instrumentation™, Brazil) and was subsequently processed in an analog-digital module (USB-6008^®^ signal-conditioning module, National Instruments^TM^, Austin, TX, USA) to be recorded in LabView (Signal Express 2009, National Instruments^TM^, USA) at 30 Hz. These mV signals are related to the weights of the calibration loads and converted to mass units in grams (g). The system was previously calibrated using 9 loads from 0.198 to 7.938 kg. The signs were treated using MatLab^®^ 7.0 (MathWorks™) according to Scariot et al. [61]. Spontaneous physical activity recordings were performed for 24 continuous hours (on non-training days), being started at 6:00 p.m. Given that single housing (in order to obtain individual values) could be deleterious by disturbing daily routines of animals, we measured spontaneous physical activity of rats on a per-cage basis, as they are normally maintained (5 rats per cage). The daily spontaneous physical activity was determined by the sum of variations of weight, in absolute values, according to a mathematical strategy proposed by Biesiadecki et al. [62].

### 4.7. Obtaining and Storing Biological Material

After the 12-week experiment, euthanasia was performed by decapitation, a method that can be used according to the American Veterinary Medical Association [63], 48 h after the last physical training session (to avoid any acute effect of exercise on experimental variables). The animals were not fasted before blood collection. The blood sample was collected from the region of the body at which the animal was decapitated. Immediately after collection, the blood rested for 15 min (4 °C) and then was centrifuged for 15 min at 3000 rpm for serum separation and blood glucose analysis. Subsequently, soleus muscle was excised, dusted in talcum, frozen in liquid nitrogen (−196 °C) and stored at −80 °C until the histological procedures. The tissues collected were soleus, gluteus maximus and gastrocnemius skeletal muscle for glycogen analysis. For gluteus maximus and gastrocnemius, levels of triacylglycerol were also determined.

### 4.8. Histology and Immunofluorescence Procedures

The protein expressions of this study were performed by immunofluorescence [64] and the technique performed according to Faria et al. [65] and Pejon et al. [66]. The skeletal muscle evaluated was the soleus due to the high recruitment during the performance of the swimming exercise [32]. Firstly, transversal histological frozen sections (6 μm) of soleus muscles were obtained from a cryostat (Leica CM 1850 UV) at −25 °C and collected in glass slides. The slides were stained by hematoxylin-eosin (HE) to identify morphological alteration in tissue through a light microscope. Three tissue sections were collected from each animal and then prepared in separate slides for each image.

For GLUT4 quantification, tissue sections were double stained with laminin and anti-GLUT4. Briefly, slides were incubated with a mix of the primary antibodies anti-GLUT4 (mouse monoclonal, dilution 1:1600; Santa Cruz Biotechnology, Inc.; Dallas, TX, USA) and anti-laminin (rabbit, dilution 1:200; Ab11575; Abcam; Cambridge, UK) diluted in BSA 1% (Bovine Serum Albumin—Sigma Aldrich Chemical Corporation, St Louis, MO, USA) for 45 min at 37 °C. Then, the sections were washed in PBS solution (total of 15 min in 3 cycles) and incubated by a mix of secondary antibodies: Alexa 488 IgG^1^ to mark GLUT4 in green color (dilution 1:1000; Jackson ImmunoResearch Laboratories, Inc.; West Grove, PA, USA) and Alexa Fluor 647 IgG to mark laminin in red color (dilution 1:200; Invitrogen; Life Technologies; Carlsbad, CA, USA) for 35 min at 37 °C. The sections were washed again with PBS solution.

The same staining procedure was used for FAT CD36. A mix of the primary antibodies anti-FAT CD36 (mouse monoclonal, dilution 1:400; Santa Cruz Biotechnology, Inc.; Dallas, TX, USA) and anti-laminin (rabbit, dilution 1:200; Ab11575; Abcam; Cambridge, UK) was applied on sections for 45 min at 37 °C. Secondary antibodies used were Alexa 594 IgM to mark FAT CD36 in red color (dilution 1:1000; Jackson ImmunoResearch, Laboratories, Inc.; West Grove, PA, USA) and Alexa Fluor 488 IgG to mark laminin in green color (diluted 1:200; Invitrogen; Life Technologies; Carlsbad, CA, USA). The slides were photographed in an automated fluorescence microscope system (ImageXpress^®^ Micro, Molecular Devices) using a 20x objective lens with specific filters according to the employed fluorophores: GLUT4/laminin (FITC/Cy5; 1000–1200 ms exposure/200 ms exposure) and FAT CD36/laminin (Cy5/FITC; 100-2200 ms exposure/200 ms exposure). All images were saved at exactly the same size and resolution to avoid decreasing the image quality. After image acquisition, five standardized areas (height: 220 and width:220) of each section (three sections per animal) were randomly chosen for further quantification of proteins using ImageJ 1.52a software (National Institutes of Health, USA). The images and fields were randomly selected, the images analyzed without overlay (without laminin marking as observed in the Figure 1) and the fields with standardized background containing exclusively each protein of interest (green dots: GLUT4 and red dots: FAT CD36). The average value of integrated intensity obtained by software was used for the graph plot.

### 4.9. Glycogen

The technique followed the protocol of Dubois et al. [67]. Briefly, skeletal muscle (200–250 mg) was digested in potassium hydroxide (KOH 30%; Cod. HP09874RA; Êxodo Científica) and glycogen was precipitated using sodium sulfate (100 µL, Na_2_SO_4_; Cod. P.10.0642.000.00; Dinâmica Química Contemporânea LTDA) and ethanol (3 mL, CH_3_CH_2_OH, 70%; Cod. QHA010; QHEMIS). From a chemical reaction using phenol (10 µL, C_6_H_5_OH; Cod. FC09662PA; Êxodo Científica) and sulfuric acid (2.0 mL, H_2_SO_4_; Cod. 31190; Synth), a glycogen concentration was colorimetrically measured by the sample absorbance. The reading was made in a spectrophotometer (Hach Company, Loveland, CO, USA) with a wavelength of 490 nm.

### 4.10. Muscular Triglyceride

First, 1 mL of Triton X-100 was added at 0.1% for every 200 milligrams of muscular tissue. It was subsequently mechanically homogenized and centrifuged for 10 min at 4000 rpm. Then, 10 μL of the supernatant was collected from each sample and pipetted into a microplate with 200 μL of reagent from the commercial triacylglycerol kit (Cod. 1770290; LaborLab; Guarulhos, SP, Brazil) and incubated for 20 min (25 °C): good (pH = 6.8, 50 mmol/L), chlorophenol (2 mmol/L), lipoprotein lipase (≥ 800 U/L), GK (≥ 500 U/L), GPO (≥ 1500 U/L), POD (≥ 900 U/L), ATP (2 mmol/L) and 4-AF (0.4 mmol/L). The reading was conducted using a spectrophotometer SpectraMax i3 (Molecular Devices) with a wavelength of 505 nm, following the kit guidelines.

### 4.11. Blood Glucose Analysis

In each serum sample containing 3 μL, 300 μL of reagent was added from a commercial glucose kit (Cod. 1770130; LaborLab; Guarulhos, SP, Brazil) and incubated for 25 min (25 °C): GOD (≥ 15 kU/L), POD (≥ 2 kU/L), 4-AAT (0.5 mmol/L), phosphates (pH = 7.5, 250 mmol/L) and phenol (5 mmol/L). The reading was conducted using spectrophotometer SpectraMax i3 (Molecular Devices) with a wavelength of 505 nm, following the kit guidelines.

### 4.12. Statistical Analysis

The results were presented as mean ± standard deviation. Data were submitted to Shapiro–Wilk’s normality test, allowing parametric statistics usage. Data from FAT CD36 and GLUT4, glycogen, muscular triglyceride and blood glucose were subjected to a two-way factorial analysis of variance for the main effects of physical training (PT, two levels: CG and OG vs. EG and OEG) and ovariectomy (OVX, two levels: CG and EG vs. OG and OEG). Spontaneous physical activity and body mass were registered multiple times over 12 weeks by being analyzed by three-way factorial to evaluate the effects of the interventions (PT and OVX) and time. When appropriate, we used the Newman–Keuls post hoc test. A significance level of 5% was established for all analysis, and Statistica 7.0 (StatSoft, Inc.) was used. The effect sizes were calculated according to Cohen [68] and the lowest values found were FAT CD36 = 1.80 (CG vs. EG); GLUT4 = 1.26 (CG vs. OG); blood glucose = 1.61 (EG vs. OEG); body mass = 1.05 (CG vs. OEG in the 7th week).

## Data Availability

Data are contained within the article.
